# Vest Pocket Anatomist

**Published:** 1887-10

**Authors:** 


THE VEST POCKET ANATOMIST (*founded upon Gray*). By C. Henri Leonard, A. M., M. D., Professor of the Medical and Surgical Diseases of Women in the Detroit College of Medicine. 13th Revised Edition. Detroit: The Illustrated Medical Journal Co. 1887.

To a medical student it is indispensable. Properly used, it is a valuable possession. H. H.

